# Integrating biomarkers and multi-parametric MRI to provide enhanced clinical diagnosis for prostate cancer

**DOI:** 10.3389/fruro.2023.1235944

**Published:** 2023-07-17

**Authors:** Jason Alter, David M. Albala

**Affiliations:** ^1^ Department of Clinical and Scientific Affairs, Exosome Diagnostics, Inc., Waltham, MA, United States; ^2^ Urology, Associated Medical Professionals, Syracuse, NY, United States; ^3^ Department of Urology, Downstate Health Sciences University, Brooklyn, NY, United States

**Keywords:** mpMRI, biomarker, clinical management, risk assessment, prostate cancer

## Abstract

Prostate cancer (PCa) risk assessment can incorporate clinical features, gene expression, protein ‘biomarkers’ or imaging. In this review the benefits of layering multiparametric magnetic resonance imaging (mpMRI) with other risk assessment methods is considered. mpMRI is an increasingly utilized risk assessment tool in prostate cancer. The European Association of Urology, National Comprehensive Cancer Network (NCCN) and American Urological Association (AUA) guidelines call for mpMRI utilization in the prostate cancer management pathway. As such, the NCCN Guidelines and AUA guidelines emphasize differing levels of reliance on mpMRI preceding prostate biopsy. However, like all risk assessment tools, mpMRI has strengths and limitations. This include dependencies on reader expertise and interpretation, equipment and process standardization, tumor size, tumor multifocality, tissue architecture, ethnic and racial disparity, and cost. Thus, layering complementary risk assessment methods to mitigate the limitations of each approach, enables the most informed clinical management. The goal of ongoing biomarker/mpMRI studies is to provide insight into the clinically helpful integration of the two approaches. For new technologies to be adapted or layered together synergistically, five specific competencies must be considered acceptable: (1) efficacy, (2) potential side effect levels, (3) ease of use of technology, (4) cost vs. clinical benefit, and (5) durability.

## 1 Introduction

Prostate cancer (PCa) is a leading cause of cancer death among men in the United States. In 2023, 288,300 new prostate cancer diagnoses are projected with an expected mortality of ~34,700. Prostate needle biopsies are recommended for men with elevated prostate-specific antigen (PSA) levels and/or a suspicious digital rectal exam (DRE) with added considerations based on family history, age, and race (1). Prostate biopsy outcomes are often benign tissue or an over-diagnosis of low-grade disease (Gleason grade group [GG] 1) and largely results in active surveillance. Additionally, shared decision-making for prostate biopsy using standard of care does result in missed high-grade prostate cancer (HGPC) (2), which is concerning given the disturbing increase in distant metastasis (3). There is a key need to integrate diagnostic approaches to better inform biopsy decisions for high-risk men, while deferring biopsy for low-risk men. This literature review was conducted using the following search term groupings: MRI, prostate; biomarkers, prostate, MRI; and prostate, MRI, NPV. Here, we highlight how biomarkers and multi-parametric magnetic resonance imaging (mpMRI) are complementary diagnostic tools that could provide synergistic clinical value.

### 1.1 mpMRI in prostate cancer

mpMRI is a powerful technology that provides insight into which patients may harbor clinically concerning tumors. As with all risk assessment methodologies, mpMRI has strengths and limitations. Comparing mpMRI performance across studies is difficult due to variations in study design, equipment (e.g., magnet strength), inconsistent incorporation of the three individual phases (T2 weighted, diffusion, and dynamic contrast-enhanced), as well as reader expertise. Similarly, biopsy methodology varies greatly as do the types of biopsy samples evaluated (4–7). These ‘mixed use’ inclusion criteria impact disease prevalence, which affects both positive and negative predictive values (8). Inconsistencies in the definition of HGPC across mpMRI studies can also skew results. While many use the HGPC definition provided by the International Society of Urological Pathology (ISUP) of ≥GG2, other definitions include ISUP grade ≥GG3, core length, positive cores percentage or some combination (9–15). How HGPC definitions and biopsy methodology vary and impact HGPC incidence has been summarized previously (16).

The mpMRI HGPC detection metrics depend upon the selected biopsy method (transrectal ultrasound scan (TRUS)-guided, targeted, etc.) (**Table 1**). Studies still vacillate on the most appropriate use of TRUS-guided biopsy versus targeted biopsy (7, 17, 23). Though often debated, it is accepted that mpMRI imaging provides equal or superior detection over TRUS-guided biopsy (18, 24). NCCN guidelines call for the ‘routine’ use of image-guided biopsy, but also highlight the potential value of a systemic biopsy in addition to image guided biopsy (1). Recently updated American Urology Association (AUA) guidelines call for a TRUS-guided vs targeted biopsy based on Prostate Imaging Reporting & Data System (PI-RADS) scores (25).

**Table 1:** Detection of Prostate Cancer in Positive mpMRI (≥PI-RADS 3).

Clinical Trial	N	Gleason Grade Group (GG) and lesion detection method					
		GG≥2 Detected mpMRI TBx	GG≥2 Detected TRUS Bx	GG1 Detected mpMRI TBx	GG1 Detected TRUS Bx	GG≥2 Combined TBx & TRUS	GG1 Combined TBx & TRUS
Prospective (17)	1042	28%	24%	16%	25%	35%	25%
PRECISION (18) (NCT02380027)	500	38%	26%	9%	22%	––	––
Single site (19)	343	––	––	––	––	57%	12%
PAIRED CAP (13) (NCT02425228)	300	76%	77%	24%	23%	70%	––
MRI-FIRST (20) (NCT02485379)^*^	251	32%	27%	5.6%	20%	35%	22%
Retrospective (21)	560	BxNaive 36% PriorNeg 28%	BxNaive 34% PriorNeg 26%	BxNaive 15% PriorNeg 9%	BxNaive 27% PriorNeg 23%	BxNaive 44%	––
Retrospective (22)^*,**^	640	48.4%	––	15.2%	––	49.8%	15.5%^**^
PRECISE (23) (NCT02936258)	453	35%	30%	10%	22%	––	––
NCT03377881 (24)	1532	––	18%	––	12%	21%	4%

*In this analysis, PI-RADS1-5 biopsy data was provided, but metrics were analyzed only for a definition of HGPC of >GG2. PI-RADS >3 was considered MRI positive in this table. **In this study, TRUS categories refer to non-targeted biopsies.

GG, Gleason grade group; Bx, biopsy; BxNaive, initial biopsy; mpMRI, multi-parametric magnetic resonance imaging; MRI, magnetic resonance imaging; Neg, negative; PI-RADS, Prostate Imaging Reporting and Data System; PriorNeg, repeat biopsy for prior negative biopsy result; TBx, targeted biopsy; TRUS, transrectal ultrasound scan.

### 1.2 Factors affecting mpMRI tumor detection

The most widely acknowledged factors that impact mpMRI-specific tumor detection are reader interpretation, reporting, and biopsy methodology (26).

### 1.3 Reader variability/subjectivity and PI-RADS variation

PI-RADS scores are a group-based risk assessment that provides the probability, not guarantee, of a biopsy result (10). A key foundation of PI-RADS is a critical mpMRI limitation, reader-dependent variation. Although high concordance (78%) is claimed in studies that utilize ‘expert’ readers, the definition of ‘expert’ raises concerns surrounding the use of generalizations in clinical practice (18, 27, 28). A recent study spanning 26 sites highlighted that generalization of mpMRI led to varying positive predictive values (PPV) between sites due to reader variation, poor targeting, and inconsistent disease prevalence (29). Thus, there is a need for mpMRI standardization.

## 2 A fundamental limitation is mpMRI’s negative predictive value

Previous studies indicate that mpMRI appears to be better at finding larger, solitary tumors than multi-focal or smaller tumors (14, 30–32). While mpMRI PPV does vary, it becomes less so as PI-RADS scores increase (13, 18, 26). False positive MRI readings can be caused by conditions such as hyperplasia, inflammation, fibrosis, prostatitis, and high-grade prostatic intraepithelial neoplasia (HGPIN) (33). However, perhaps more concerning is mpMRI’s negative predictive value (NPV). This is largely because a negative result often leads to the clinical decision to defer a biopsy (17). Tumor size, grade, multifocality, tissue architecture, and gene expression affect tumor visibility and caution is advocated when interpreting mpMRI negative results (1, 34–36). Moreover, up to 35% of HGPC tumors are not visible on mpMRI (17, 32) and HGPC (≥GG2) is often found after a negative mpMRI (9, 26, 27, 37). Thus, NCCN guidelines suggest caution when assessing negative mpMRI results and newly updated AUA guidelines call for TRUS biopsy for PI-RADS <3 and targeted biopsy for PIRADS 3-5 (1, 25).

**Figure 1:**
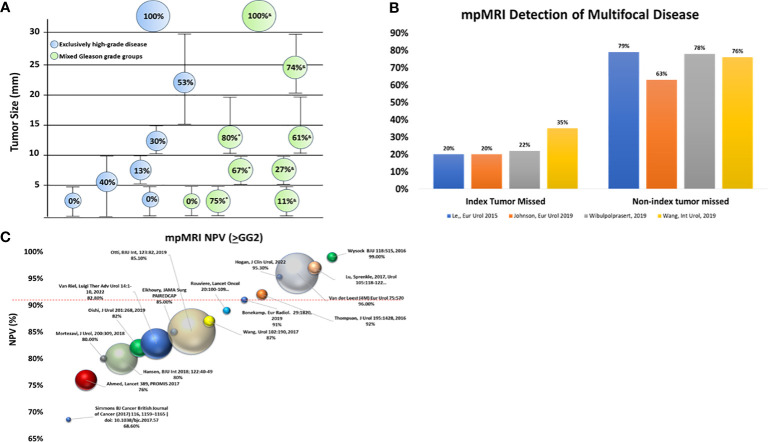
Cancer detection with mpMRI. **(A)** The impact of tumor size on mpMRI tumor detection. Bubble size reflects the percentage (%) of tumors detected by mpMRI: larger bubbles indicate a higher detection rate than smaller bubbles. Green bubbles are mixed Gleason grade groups while blue bubbles are exclusively high-grade disease (≥GG2) (30, 31, 36, 38, 39). **(B)** Ability of mpMRI to detect multi-focal disease. Although mpMRI misses the index lesion 20%-30% of the time, non-index lesions are missed much more frequently (63%-79%) (21, 31, 32, 39). **(C)** References reviewed in Moldavan et al. (8) are excluded except for the PROMIS study (9). All NPVs reported for HGPC are defined as ≥GG2 unless a specific notation is included. Ball location on the Y-axis indicates NPV, while ball size indicates cohort size for mpMRI results < PI-RADS 3. GG, Gleason grade group; HGPC, high grade prostate cancer; mpMRI, multi-parametric magnetic resonance imaging; MRI, magnetic resonance imaging. NPV, negative predictive value. *Sizes based on solidarity tumors; &Sizes based on multifocal tumors.

Tumor size is important to mpMRI detection (**Figure 1A**). Although mpMRI can miss tumors >1 cm, 43% to 82% of tumors <1 cm are invisible on mpMRI (31). Studies incorporating radical prostatectomy (RP) provide pathologic truth on the association between tumor size/lesion focality and mpMRI detection (30–32), with tumor detection decreasing as tumors become smaller. This is problematic as tumors <6 mm in size, can harbor high-grade disease (32, 38).

Tumor location is also important in mpMRI detection. Lesions are found in all prostate zones (peripheral, transition, and anterior) (6, 17) and mpMRI false negatives can occur in all zones (10). MRI-targeted biopsy (MRI Tbx) compared with TRUS-guided biopsy observed a 64% concordance in tumor detection in the same prostate zone. The remainder of tumors were detected by only one type of biopsy methodology, suggesting that different biopsy approaches find different tumors (13). Proximity to the prostate capsule also correlates with MRI visibility with tumors ≤0.05 cm from the capsule detected more often (46%) than >0.5 cm away (16.7%) (30).

PCa multifocality is well established with separate foci displaying both Gleason score and genomic heterogeneity (40). Multifocality increases the probability that mpMRI miss tumors (**Figure 1B**) (19, 32, 39). In a study of follow-up fusion biopsy with a TRUS-guided biopsy revealed 30% HGPC outside the index lesion, with the Gleason score being greater than or equal to that found in index lesions. The risk of finding HGPC outside the MRI-located index lesion increased as the PI-RADS score increased, with a 10% probability for PI-RADS 2, which rose to a 70% probability for PI-RADS 5 (19). Whole mount RP studies show that index tumors are more easily detected than non-index lesions (32, 38, 39). Moreover, although MRI-detected HGPC lesions in 97% of patients, additional PI-RADS ≥3 lesions were missed in 60% of these cases (7).

### 2.1 Negative predictive values

Analysis of studies with mpMRI negative results (PI-RADS <3) demonstrate that mpMRI NPV is quite variable (1, 4, 5, 7, 9–15, 26). Of particular importance, Otti et al. found that 17% of men with ‘normal’ mpMRI readings had palpable disease (26). Chung et al. examined ‘invisible’ tumors (PI-RADS <3) with biopsy and RP, finding at biopsy that 24% were ≥GG2 and 6.6% were ≥GG4 (41). Finally, men initially classified as mpMRI negative at 2.4 years post-imaging identified the false negative rate to be 23% for HGPC (42).

Smaller cohort studies frequently present NPV and PPV, often combining different biopsy sample types (biopsy naive, prior negative, active surveillance) without sufficient regard to the impact on disease prevalence (4–8, 15). Although some studies conduct sub-analysis to examine metrics separately based on biopsy type, many do not (5, 9, 16). In 2017, Moldovan et al. (8) performed a thorough review and documented the impact of clinical heterogeneity and disease prevalence on NPV. Therefore, with the exception of the landmark PROMIS trial (9), only studies published since Moldovan et al. (8) are reviewed here (**Figure 1C**). Unfortunately, if a study did not biopsy mpMRI negative results (<PIRADS 3) it could not be included in the analysis (including the otherwise well-executed PRECISION trial (18)).

Since the Moldovan review (including PROMIS), there have been 2,035 mpMRI negative cases (PI-RADS 1/2s) across these studies. As shown in **Figure 1C**, a number of studies with mixed biopsy types (4, 6, 7, 15, 16), small sample sizes, and/or solitary experienced readers trend towards higher NPVs (4, 5, 12, 43). The majority (68%) of these studies resulted in NPV <90% with the landmark PROMIS trial observing 76% NPV for ≥GG2. Only 32% (N=653) of the data generated NPVs ≥90%. In fact, the best study for initial biopsy with NPV above 90%, comprising 47% (N=309) of the cases with NPV >90%, was the 4M study (96%) (44). This prospective trial employed trained radiologists, centralized image review and consensus assessment resulting in an atypical low number of PI-RADS 3 (6%). Due to the expertise in this study, the generalizability of the results to routine practice is a legitimate question. Indeed, the authors believe a key limitation of their data is its performance reproducibly outside their ‘expert’ sites (44).

In addition to sample size and range of mpMRI protocols, biopsy methodology also limits mpMRI performance metrics. In particular, the widely used TRUS-guided biopsy provides an imperfect window into mpMRI performance. Studies that utilize more holistic assessment methods such as template biopsy mapping, saturation biopsy, or whole mount RP offer the most comprehensive pathologic ground truth for assessing performance metrics (9, 10, 14, 32, 45, 46). The landmark PROMIS study arguably provides the most comprehensive assessment of mpMRI metrics because it employed template biopsies for all PI-RADS categories (9). Although the 1.5 Tesla (T) magnet strength in the PROMIS study was less than the often employed 3T magnet strength, much of the essential mpMRI literature utilizes a mix of magnet strengths (both 1.5T and 3.0T) (18, 20, 24). Furthermore, some studies have found no difference in PPV or NPV when comparing 1.5T or 3T generated data (11, 29).

The PROMIS study combined high quality and standardized MRI, in-depth reporting, dedicated and experienced urologic radiologists, centralized reader training, TRUS biopsies, and high-quality targeted mapping biopsies every 5 mm. The primary definition of clinically significant cancer was defined as ≥GG3 or cancer core length ≥6 mm, but two additional cancer definitions were also measured (1) ≥GG2 or (2) cancer core length ≥4 mm. As such, each definition resulted in different performance metrics. The primary definition of ≥GG3 or cancer core length ≥6 mm had a PPV of 51% and NPV of 89%. The more widely used definition, ≥GG2, had a PPV of 65% and an NPV of 76% (9). Other studies utilizing mapping biopsies have demonstrated similar NPV for ≥GG2 or related definitions. Simmons et al. noted an NPV of 68.6 for ≥GG2 and/or tumor length of ≥4 mm (14). Mortezavi et al. used template saturation biopsy to measure mpMRI performance metrics for ≥GG2 and noted overall NPVs of 74.2% and 68.5% for the saturation biopsy and targeted fusion biopsy, respectively. The authors conducted a subgroup analysis according to biopsy type (naïve, prior negative biopsy, or positive biopsy), resulting in changed predictive values due to changes in disease prevalence (16).

### 2.2 Biomarkers and mpMRI

Considerations for adding biomarker information to mpMRI has clinical benefit, with the reduction of potential side effects, ease of use, cost, and ability for generalizations. Evaluating biomarker data can be challenging because there is no one-size-fits-all approach and biomarkers will fit each case differently. The advantages are that biomarkers for early detection of PCa can be analyzed in urine, blood, or post-needle biopsy samples (47–50). One test already has a home collection kit further simplifying sample collection even further (51). When considering deferred HGPC as a potential side effect of early detection risk assessment, the percentage of deferred HGPC depends upon the biomarker threshold utilized.

Biomarker durability and generalizability vary due to biomarker study design ranging from observational and retrospective to prospective clinical trials. In prospective studies for the intended use population, biomarker durability and clinical benefit are evident (52). However in retrospective analyses, when cohorts do not represent the intended population, it is difficult to assess the actual performance or generalizability of the biomarker (48, 49, 53). Furthermore, most biomarkers include clinical information combined with genomic data making it difficult to determine the specific value the unique test components provide when not supported by the clinical features (48, 49, 53). However, stand-alone biomarker assays do exist that do not integrate clinical features and have been used in studies with prospective trial design, tailored to intended-to-use population (47, 54).

Non-invasive tests such as PSA density, risk calculators, genomic testing, and commercial biomarker tests all appear to provide some degree of enhanced clinical risk assessment when appropriately layered into a clinical pathway with mpMRI (6, 13, 15, 37, 45). PSA density (PSAD), for example, is often found to be complementary to mpMRI (6, 12, 13, 37, 55, 56). In one study, 63% of men with abnormal mpMRI results and HGPC had a PSAD of ≥0.15 ng/mL, compared to 29% of men with normal mpMRI readings and benign or insignificant PCa who had PSAD of ≤0.15 ng/mL (26). Another study observed that PSAD >0.1 ng/mL provides complementary risk assessment value to mpMRI for HGPC defined as ≥GG2. Furthermore, an increased frequency of PI-RADS 4 and 5 lesions with HGPC was observed when PSAD was above the 0.15 ng/mL threshold (56). Conversely, other research has shown that when PI-RADS is low-risk (≤2) a PSAD <0.15 ng/mL may improve the NPV (12). In a mixed group of biopsy naïve men (36%) and men with a prior negative biopsy and a low-risk mpMRI (PI-RADS <3), mpMRI NPV improved from 82% to 90% with a PSAD <0.15 ng/mL. In solely biopsy naive men with negative mpMRI and PSAD <0.15 ng/mL, the NPV improved from 71% to 80% (6). Moreover, the mpMRI NPV for prostate biopsy naïve men with PI-RADS 1 or 2 was 80% that increased to 91% when PSAD <0.1 ng/mL was included in a prospective study (57). Other studies have specifically focused on the clinical benefit of combining PSAD and mpMRI for PI-RADS 3 scores with HGPC detection across PI-RADS scores gated by PSADs of <0.15, 0.15-0.29, or ≥0.3 ng/mL. The highest HGPC detection rate (97%) was found when PI-RADS 4 and 5 had a PSAD of ≥0.3 ng/mL. In contrast, the lowest HGPC detection rate (0%) was found in PI-RADS 1 and 2 scores with PSAD <0.15 ng/mL and no HGPC was detected in PI-RADS 3 with PSAD <0.15 ng/mL (N=6). Although the numbers were small, the authors conclude that men with a PI-RADS score ≤3 and PSAD <0.15 ng/mL may be able to avoid unnecessary biopsies (37).

Beyond individual biomarkers, the next step is to assess how integrating multiple biomarkers might complement mpMRI. The current landscape is expanding PSAD to numerous clinical features and mpMRI (15), with clinical feature calculators being integrated to mpMRI interpretation. Initial results of these calculators are highlighting that some clinical risk features are more important to NPV than others (15).

Although published studies have limitations, the biomarker/mpMRI literature suggests that commercial biomarker tests and mpMRI capture independent information that can provide a synergistic benefit (58). Several biomarker studies have demonstrated a correlation between increasing PI-RADS scores and biomarker scores, and suggest that performance for HGPC detection improves when mpMRI and biomarkers are combined (49, 59). An observational study concluded that the SelectMDx biomarker had independent information that improved the PI-RADS area under the curve (AUC). However, the picture was incomplete since PI-RADS <3 biopsies were not included (60). Another biomarker, Proclarix, combines clinical features with thrombospondin-1 and cathepsin, which when combined with mpMRI, the biomarker demonstrated improved performance and increasing mpMRI NPV up to 6% in men with PI-RADS <3 (61). The actual biomarker value versus the clinical feature performance in these studies is unclear as the initial and repeat biopsies were mixed. Moreover, the HGPC prevalence in one study was lower than in an intended-to-use population. The Myprostatescore biomarker improved NPV in men with PI-RADS 3 and performed better than PSA density. However, 57% of the cohort had a prior negative biopsy, lowering the HGPC prevalence and likely artificially inflating NPV (48). Preliminary data with the ExoDx Prostate biomarker and mpMRI has shown an association between rising ExoDx Prostate IntelliScore (EPI) scores and PI-RADS, demonstrating the benefit of modeling EPI and mpMRI together (59). Specifically, the PCA3 biomarker and mpMRI combination in men going for initial biopsy had an improved performance over mpMRI alone (62). A superior performance in AUC was observed when combining the 4K biomarker with mpMRI, each providing independent and complementary information (53). Similarly, a complementary performance was detected when combining PSA density, the prostate health index (PHI) biomarker, and mpMRI for men with a prior negative biopsy (63). Finally, a prospective non-inferiority trial demonstrated that a clinical workflow combining the Stockholm biomarker and mpMRI detected more HGPC and fewer low-grade cancers (64).

The cost of integrating biomarkers with mpMRI is variable based on the biomarker. mpMRI is an expensive procedure with a median cost of $4396 (interquartile range $2,784-$7,127) for MRI-guided biopsy, increasing to $5,832 when anesthesia is used (65). The cost of mpMRI/biomarker care will likely depend on how the two modalities are implemented. In risk assessment for early PCa detection, multiple clinical combinations of commercial biomarkers and mpMRI have been presented (66), with initial data suggesting biomarkers placed before mpMRI will provide the most clinical benefit (50). Additional cost savings may also result from reducing un-needed biopsies and reducing the use of mpMRI for men at low risk for finding HGPC (67).

## 3 Conclusion

All risk assessment methods, including mpMRI and biomarkers, have strengths and limitations. The adoption of mpMRI in the urology field must be balanced with enhanced education and training on the strengths and limitations of the technology. How biomarkers can be appropriately integrated is also imperative. The ReIMAGINE Consortium was explicitly established to develop risk assessment tools that can examine the benefits of combining mpMRI with biomarkers (68). Guidelines reflect a careful view of the existing data and emphasize that a negative mpMRI does not omit the possibility of cancer. Moreover, clinicians should consider biomarkers when looking to defer a biopsy in a patient with a negative mpMRI (1, 25). Specific biomarkers not only have good performance in prospective clinical trials, but also offer significant logistical advantages that complement mpMRI utilization. Non-invasive urinary biomarkers have clear logistical benefits (52, 67), as some do not require a DRE and urine collection can occur in the clinic or a patient’s home (51). Taken together, biomarkers should have a complementary role to mpMRI. As mpMRI utilization grows, biomarker use will grow in parallel. It is imperative to understand how to integrate the two technologies appropriately to enhance clinical practice.

## Author contributions

All authors contributed to the conception and design, manuscript revisions, and approved the final version of the manuscript.

## References

[1] Schaeffer EM Srinivas S Adra N An Y Barocas D Bitting R. NCCN guidelines^®^ insights: prostate cancer, version 1.2023. J Natl Compr Canc Netw (2022) 20:1288–98. doi: 10.6004/jnccn.2022.0063 36509074

[2] Tutrone R Donovan MJ Torkler P Tadigotla V McLain T Noerholm M. Clinical utility of the exosome based ExoDx Prostate(IntelliScore) EPI test in men presenting for initial biopsy with a PSA 2–10 ng/mL. Prostate Cancer Prostatic Dis (2020) 23:607–14. doi: 10.1038/s41391-020-0237-z PMC7655505 32382078

[3] Jemal A Culp MB Ma J Islami F Fedewa SA. Prostate Cancer Incidence 5 Years After US Preventive Services Task Force Recommendations Against Screening. J Natl Cancer Inst (2021) 113:64–71. doi: 10.1093/jnci/djaa068 PMC7781461 32432713

[4] Wysock JS Mendhiratta N Zattoni F Meng X Bjurlin M Huang WC. Predictive value of negative 3T multiparametric magnetic resonance imaging of the prostate on 12-core biopsy results. BJU Int (2016) 118:515–20. doi: 10.1111/BJU.13427 26800439

[5] Wang RS Kim EH Vetter JM Fowler KJ Shetty AS Mintz AJ. Determination of the role of negative magnetic resonance imaging of the prostate in clinical practice: is biopsy still necessary? Urology (2017) 102:190–7. doi: 10.1016/j.urology.2016.10.040 27845218

[6] Oishi M Shin T Ohe C Nassiri N Palmer SL Aron M. Which patients with negative magnetic resonance imaging can safely avoid biopsy for prostate cancer? J Urol (2019) 201:268. doi: 10.1016/J.JURO.2018.08.046 30189186 PMC6677264

[7] Bonekamp D Schelb P Wiesenfarth M Kuder TA Deister F Stenzinger A. Histopathological to multiparametric MRI spatial mapping of extended systematic sextant and MR/TRUS-fusion-targeted biopsy of the prostate. Eur Radiol (2019) 29:1820–30. doi: 10.1007/S00330-018-5751-1/METRICS 30327861

[8] Moldovan PC Van den Broeck T Sylvester R Marconi L Bellmunt J van den Bergh RCN. What is the negative predictive value of multiparametric magnetic resonance imaging in excluding prostate cancer at biopsy? a systematic review and meta-analysis from the European association of urology prostate cancer guidelines panel. Eur Urol (2017) 72:250–66. doi: 10.1016/J.EURURO.2017.02.026 28336078

[9] Ahmed HU El-Shater Bosaily A Brown LC Gabe R Kaplan R Parmar MK. Diagnostic accuracy of multi-parametric MRI and TRUS biopsy in prostate cancer (PROMIS): a paired validating confirmatory study. Lancet (2017) 389:815–22. doi: 10.1016/S0140-6736(16)32401-1 28110982

[10] Borofsky S George AK Gaur S Bernardo M Greer MD Mertan FV. What are we missing? false-negative cancers at multiparametric MR imaging of the prostate. Radiology (2017) 286:186–95. doi: 10.1148/RADIOL.2017152877 PMC5749595 29053402

[11] Thompson JE Van Leeuwen PJ Moses D Shnier R Brenner P Delprado W. The diagnostic performance of multiparametric magnetic resonance imaging to detect significant prostate cancer. J Urol (2016) 195:1428–35. doi: 10.1016/J.JURO.2015.10.140 26529298

[12] Hogan D Yao HHI Kanagarajah A Ogluszko C Tran PVP Dundee P. Can multi-parametric magnetic resonance imaging and prostate-specific antigen density accurately stratify patients prior to prostate biopsy? J Clin Urol (2022) 1–8. doi: 10.1177/20514158221084820

[13] Elkhoury FF Felker ER Kwan L Sisk AE Delfin M Natarajan S. Comparison of targeted vs systematic prostate biopsy in men who are biopsy naive: the prospective assessment of image registration in the diagnosis of prostate cancer (PAIREDCAP) study. JAMA Surg (2019) 154:811–8. doi: 10.1001/JAMASURG.2019.1734 PMC6563598 31188412

[14] Simmons LAM Kanthabalan A Arya M Briggs T Barratt D Charman SC. The PICTURE study: diagnostic accuracy of multiparametric MRI in men requiring a repeat prostate biopsy. Br J Cancer (2017) 116:1159. doi: 10.1038/BJC.2017.57 28350785 PMC5418442

[15] van Riel LAMJG Jager A Meijer D Postema AW Smit RS Vis AN. Predictors of clinically significant prostate cancer in biopsy-naïve and prior negative biopsy men with a negative prostate MRI: improving MRI-based screening with a novel risk calculator. Therapeutic Adv in Urol (2022) 14:1–10. doi: 10.1177/17562872221088536 PMC8958520 35356754

[16] Mortezavi A Märzendorfer O Donati OF Rizzi G Rupp NJ Wettstein MS. Diagnostic accuracy of multiparametric magnetic resonance imaging and fusion guided targeted biopsy evaluated by transperineal template saturation prostate biopsy for the detection and characterization of prostate cancer. J Urol (2018) 200:309–18. doi: 10.1016/J.JURO.2018.02.067 29474846

[17] Filson CP Natarajan S Margolis DJA Huang J Lieu P Dorey FJ. Prostate cancer detection with magnetic resonance-ultrasound fusion biopsy: the role of systematic and targeted biopsies. Cancer (2016) 122:884–92. doi: 10.1002/CNCR.29874 PMC4777653 26749141

[18] Kasivisvanathan V Rannikko AS Borghi M Panebianco V Mynderse LA Vaarala MH. MRI-Targeted or standard biopsy for prostate-cancer diagnosis. N Engl J Med (2018) 378:1767–77. doi: 10.1056/NEJMoa1801993 PMC9084630 29552975

[19] Stabile A Dell’Oglio P De Cobelli F Esposito A Gandaglia G Fossati N. Association between prostate imaging reporting and data system (PI-RADS) score for the index lesion and multifocal, clinically significant prostate cancer. Eur Urol Oncol (2018) 1:29–36. doi: 10.1016/J.EUO.2018.01.002 31100225

[20] Rouvière O Puech P Renard-Penna R Claudon M Roy C Mège-Lechevallier F. Use of prostate systematic and targeted biopsy on the basis of multiparametric MRI in biopsy-naive patients (MRI-FIRST): a prospective, multicentre, paired diagnostic study. Lancet Oncol (2019) 20:100–9. doi: 10.1016/S1470-2045(18)30569-2 30470502

[21] Wang NN Teslovich NC Fan RE Ghanouni P Leppert JT Brooks JD. Applying the PRECISION approach in biopsy naïve and previously negative prostate biopsy patients. Urol Oncol Semin Orig Investig (2019) 37:530.e19–530.e24. doi: 10.1016/J.UROLONC.2019.05.002 31151788

[22] Miah S Hosking-Jervis F Connor MJ Eldred-Evans D Shah TT Arya M. A multicentre analysis of the detection of clinically significant prostate cancer following transperineal image-fusion targeted and nontargeted systematic prostate biopsy in men at risk. Eur Urol Oncol (2020) 3:262–9. doi: 10.1016/J.EUO.2019.03.005 31411968

[23] Klotz L Chin J Black PC Finelli A Anidjar M Bladou F. Comparison of multiparametric magnetic resonance imaging–targeted biopsy with systematic transrectal ultrasonography biopsy for biopsy-naive men at risk for prostate cancer: a phase 3 randomized clinical trial. JAMA Oncol (2021) 7:534. doi: 10.1001/JAMAONCOL.2020.7589 33538782 PMC7863017

[24] Eklund M Jäderling F Discacciati A Bergman M Annerstedt M Aly M. MRI-Targeted or standard biopsy in prostate cancer screening. N Engl J Med (2021) 385:908–20. doi: 10.1056/NEJMOA2100852/SUPPL_FILE/NEJMOA2100852_DATA-SHARING.PDF 34237810

[25] Wei JT Barocas D Carlsson S Coakley F Eggener S Etzioni R. Early detection of prostate cancer: AUA/SUO guideline part II: prostate cancer screening. J Urol (2023) 210:54–63. doi: 10.1097/JU.0000000000003492 PMC11321723 37096575

[26] Otti VC Miller C Powell RJ Thomas RM McGrath JS. The diagnostic accuracy of multiparametric magnetic resonance imaging before biopsy in the detection of prostate cancer. BJU Int (2019) 123:82–90. doi: 10.1111/BJU.14420 29804315

[27] Greer MD Brown AM Shih JH Summers RM Marko J Law YM. Accuracy and agreement of PIRADSv2 for prostate cancer mpMRI: a multireader study. J Magn Reson Imaging (2017) 45:579–85. doi: 10.1002/JMRI.25372 PMC7900895 27391860

[28] Rosenkrantz AB Ginocchio LA Cornfeld D Froemming AT Gupta RT Turkbey B. Interobserver reproducibility of the PI-RADS version 2 lexicon: a multicenter study of six experienced prostate radiologists. Radiology (2016) 280:793. doi: 10.1148/RADIOL.2016152542 27035179 PMC5006735

[29] Westphalen AC McCulloch CE Anaokar JM Arora S Barashi NS Barentsz JO. Variability of the positive predictive value of PI-RADS for prostate MRI across 26 centers: experience of the society of abdominal radiology prostate cancer disease-focused panel. Radiology (2020) 296:76–84. doi: 10.1148/RADIOL.2020190646 32315265 PMC7373346

[30] Wang M Janaki N Buzzy C Bukavina L Mahran A Mishra K. Whole mount histopathological correlation with prostate MRI in grade I and II prostatectomy patients. Int Urol Nephrol (2019) 51:425–34. doi: 10.1007/S11255-019-02083-8/METRICS 30671889

[31] Wibulpolprasert P Raman SS Hsu W Margolis DJA Asvadi NH Khoshnoodi P. Detection and localization of prostate cancer at 3-T multiparametric MRI using PI-RADS segmentation. American J Roentgenology (2019) 212:W122–31. doi: 10.2214/AJR.18.20113 30995090

[32] Johnson DC Raman SS Mirak SA Kwan L Bajgiran AM Hsu W. Detection of individual prostate cancer foci via multiparametric magnetic resonance imaging. Eur Urol (2019) 75:712–20. doi: 10.1016/J.EURURO.2018.11.031 30509763

[33] Gold SA Hale GR Bloom JB Smith CP Rayn KN Valera V. Follow-up of negative MRI-targeted prostate biopsies: when are we missing cancer? World J Urol (2019) 37:235–41. doi: 10.1007/S00345-018-2337-0/METRICS 29785491

[34] Zhou B Xu K Zheng X Chen T Wang J Song Y. Application of exosomes as liquid biopsy in clinical diagnosis. Signal Transduct Target Ther (2020) 5:1–14. doi: 10.1038/s41392-020-00258-9 32747657 PMC7400738

[35] Li P You S Nguyen C Wang Y Kim J Sirohi D. Genes involved in prostate cancer progression determine MRI visibility. Theranostics (2018) 8:1752. doi: 10.7150/THNO.23180 29556354 PMC5858498

[36] Truong M Hollenberg G Weinberg E Messing EM Miyamoto H Frye TP. Impact of Gleason subtype on prostate cancer detection using multiparametric magnetic resonance imaging: correlation with final histopathology. J Urol (2017) 198:316–21. doi: 10.1016/J.JURO.2017.01.077 28163032

[37] Washino S Okochi T Saito K Konishi T Hirai M Kobayashi Y. Combination of prostate imaging reporting and data system (PI-RADS) score and prostate-specific antigen (PSA) density predicts biopsy outcome in prostate biopsy naïve patients. BJU Int (2017) 119:225–33. doi: 10.1111/BJU.13465 26935594

[38] Arslan A Karaarslan E Güner AL Saǧlican Y Tuna MB Özişik O. Comparison of MRI, PSMA PET/CT, and fusion PSMA PET/MRI for detection of clinically significant prostate cancer. J Comput Assist Tomogr (2021) 45:210–7. doi: 10.1097/RCT.0000000000001116 33186177

[39] Le JD Tan N Shkolyar E Lu DY Kwan L Marks LS. Multifocality and prostate cancer detection by multiparametric magnetic resonance imaging: correlation with whole-mount histopathology. Eur Urol (2015) 67:569–76. doi: 10.1016/J.EURURO.2014.08.079 25257029

[40] Boutros PC Fraser M Harding NJ De Borja R Trudel D Lalonde E. Spatial genomic heterogeneity within localized, multifocal prostate cancer. Nat Genet (2015) 47:736–45. doi: 10.1038/ng.3315 26005866

[41] Chung DY Koh DH Goh HJ Kim MS Lee JS Jang WS. Clinical significance and predictors of oncologic outcome after radical prostatectomy for invisible prostate cancer on multiparametric MRI. BMC Cancer (2018) 18:1–10. doi: 10.1186/S12885-018-4955-8 PMC6211592 30382916

[42] Kinnaird A Sharma V Chuang R Priester A Tran E Barsa DE. Risk of prostate cancer after a negative magnetic resonance imaging guided biopsy. J Urol (2020) 204:1180–6. doi: 10.1097/JU.0000000000001232 32614257

[43] Lu AJ Syed JS Nguyen KA Nawaf CB Rosoff J Spektor M. Negative multiparametric magnetic resonance imaging of the prostate predicts absence of clinically significant prostate cancer on 12-core template prostate biopsy. Urology (2017) 105:118–22. doi: 10.1016/J.UROLOGY.2017.01.048 28322902

[44] van der Leest M Cornel E Israël B Hendriks R Padhani AR Hoogenboom M. Head-to-head comparison of transrectal ultrasound-guided prostate biopsy versus multiparametric prostate resonance imaging with subsequent magnetic resonance-guided biopsy in biopsy-naïve men with elevated prostate-specific antigen: a Large prospective multicenter clinical study. Eur Urol (2019) 75:570–8. doi: 10.1016/J.EURURO.2018.11.023 30477981

[45] Ahdoot M Wilbur AR Reese SE Lebastchi AH Mehralivand S Gomella PT. MRI-Targeted, systematic, and combined biopsy for prostate cancer diagnosis. N Engl J Med (2020) 382:917–28. doi: 10.1056/NEJMOA1910038/SUPPL_FILE/NEJMOA1910038_DATA-SHARING.PDF PMC7323919 32130814

[46] Hansen NL Barrett T Kesch C Pepdjonovic L Bonekamp D O’Sullivan R. Multicentre evaluation of magnetic resonance imaging supported transperineal prostate biopsy in biopsy-naïve men with suspicion of prostate cancer. BJU Int (2018) 122:40–9. doi: 10.1111/BJU.14049 29024425

[47] McKiernan J Donovan MJ O’Neill V Bentink S Noerholm M Belzer S. A novel urine exosome gene expression assay to predict high-grade prostate cancer at initial biopsy. JAMA Oncol (2016) 2:882–9. doi: 10.1001/JAMAONCOL.2016.0097 27032035

[48] Tosoian JJ Singhal U Davenport MS Wei JT Montgomery JS George AK. Urinary MyProstateScore (MPS) to rule out clinically-significant cancer in men with equivocal (PI-RADS 3) multiparametric MRI: addressing an unmet clinical need. Urology (2022) 164:184–90. doi: 10.1016/J.UROLOGY.2021.11.033 PMC10171463 34906585

[49] Maggi M Del Giudice F Falagario UG Cocci A Russo GI Di Mauro M. Selectmdx and multiparametric magnetic resonance imaging of the prostate for men undergoing primary prostate biopsy: a prospective assessment in a multi-institutional study. Cancers (Basel) (2021) 13:2047. doi: 10.3390/CANCERS13092047/S1 33922626 PMC8122883

[50] Falagario UG Martini A Wajswol E Treacy PJ Ratnani P Jambor I. Avoiding unnecessary magnetic resonance imaging (MRI) and biopsies: negative and positive predictive value of MRI according to prostate-specific antigen density, 4Kscore and risk calculators. Eur Urol Oncol (2020) 3:700–4. doi: 10.1016/J.EUO.2019.08.015 31548130

[51] Moul J Sant G. How I use it: the exosome diagnostics (EPI) prostate cancer biomarker utility in urology and primary care - PubMed. Can J Urol (2022) 29:11224–30. 35969726

[52] McKiernan J Donovan MJ O’Neill V Bentink S Noerholm M Belzer S. A novel urine exosome gene expression assay to predict high-grade prostate cancer at initial biopsy. JAMA Oncol (2016) 2:882–9. doi: 10.1001/jamaoncol.2016.0097 27032035

[53] Punnen S Nahar B Soodana-Prakash N Koru-Sengul T Stoyanova R Pollack A. Optimizing patient’s selection for prostate biopsy: a single institution experience with multi-parametric MRI and the 4Kscore test for the detection of aggressive prostate cancer. PloS One (2018) 13:e0201384. doi: 10.1371/JOURNAL.PONE.0201384 30092002 PMC6084850

[54] Tutrone R Donovan MJ Torkler P Tadigotla V McLain T Noerholm M. Clinical utility of the exosome based ExoDx Prostate(IntelliScore) EPI test in men presenting for initial biopsy with a PSA 2–10 ng/mL. Prostate Cancer Prostatic Dis (2020) 23:607–14. doi: 10.1038/s41391-020-0237-z PMC7655505 32382078

[55] Woźnicki P Westhoff N Huber T Riffel P Froelich MF Gresser E. Multiparametric MRI for prostate cancer characterization: combined use of radiomics model with PI-RADS and clinical parameters. Cancers (Basel) (2020) 12:1767. doi: 10.3390/CANCERS12071767 32630787 PMC7407326

[56] Frisbie JW Van Besien AJ Lee A Xu L Wang S Choksi A. PSA density is complementary to prostate MP-MRI PI-RADS scoring system for risk stratification of clinically significant prostate cancer. Prostate Cancer Prostatic Dis (2023) 26:347–52. doi: 10.1038/s41391-022-00549-y 35523940

[57] Hansen NL Barrett T Lloyd T Warren A Samel C Bratt O. Optimising the number of cores for magnetic resonance imaging-guided targeted and systematic transperineal prostate biopsy. Bju Int (2020) 125:260. doi: 10.1111/BJU.14865 31306539 PMC8641376

[58] Katzendorn O von Klot CAJ Mahjoub S Tabrizi PF Harke NN Tezval H. Combination of PI-RADS score and mRNA urine test–a novel scoring system for improved detection of prostate cancer. PloS One (2022) 17:e0271981. doi: 10.1371/JOURNAL.PONE.0271981 35960727 PMC9374213

[59] Kretschmer A Skog J Fischer C Alter J Noerholm M. A combined biomarker/mpMRI approach provides enhanced clinical information prior to prostate biopsy. Am Urol Assoc (2022) 207 (supplement 5):e193. doi: 10.1097/JU.0000000000002537.08

[60] Roumiguié M Ploussard G Nogueira L Bruguière E Meyrignac O Lesourd M. Independent evaluation of the respective predictive values for high-grade prostate cancer of clinical information and RNA biomarkers after upfront MRI and image-guided biopsies. Cancers (Basel) (2020) 12:1–12. doi: 10.3390/CANCERS12020285 PMC7072157 31991591

[61] Steuber T Heidegger I Kafka M Roeder MA Chun F Preisser F. PROPOSe: a real-life prospective study of proclarix, a novel blood-based test to support challenging biopsy decision-making in prostate cancer. Eur Urol Oncol (2022) 5:321–7. doi: 10.1016/J.EUO.2020.12.003 33422560

[62] Fenstermaker M Mendhiratta N Bjurlin MA Meng X Rosenkrantz AB Huang R. Risk stratification by urinary prostate cancer gene 3 testing before magnetic resonance imaging-ultrasound fusion-targeted prostate biopsy among men with no history of biopsy. Urology (2017) 99:174–9. doi: 10.1016/j.urology.2016.08.022 27562202

[63] Druskin SC Tosoian JJ Young A Collica S Srivastava A Ghabili K. Combining prostate health index density, magnetic resonance imaging and prior negative biopsy status to improve the detection of clinically significant prostate cancer. BJU Int (2018) 121:619–26. doi: 10.1111/BJU.14098 29232037

[64] Nordström T Discacciati A Bergman M Clements M Aly M Annerstedt M. Prostate cancer screening using a combination of risk-prediction, MRI, and targeted prostate biopsies (STHLM3-MRI): a prospective, population-based, randomised, open-label, non-inferiority trial. Lancet Oncol (2021) 22:1240–9. doi: 10.1016/S1470-2045(21)00348-X 34391509

[65] Leung AK Patil D Howard DH Filson CP. Payments and patient cost sharing for prostate biopsies according to image guidance, practice site and use of anesthesia. Urol Pract (2020) 7:138–44. doi: 10.1097/UPJ.0000000000000073 37317428

[66] Vickers AJ Russo G Lilja H Evans C Schalken JA Klein E. How should molecular markers and magnetic resonance imaging be used in the early detection of prostate cancer? Eur Urol Oncol (2022) 5:135–7. doi: 10.1016/j.euo.2021.01.010 33608234

[67] de la Calle CM Fasulo V Cowan JE Lonergan PE Maggi M Gadzinski AJ. Clinical utility of 4Kscore^®^, ExosomeDx™ and magnetic resonance imaging for the early detection of high grade prostate cancer. J Urol (2021) 205:452–60. doi: 10.1097/JU.0000000000001361 32897802

[68] Marsden T McCartan N Brown L Rodriguez-Justo M Syer T Brembilla G. The ReIMAGINE prostate cancer risk study protocol: a prospective cohort study in men with a suspicion of prostate cancer who are referred onto an MRI-based diagnostic pathway with donation of tissue, blood and urine for biomarker analyses. PloS One (2022) 17:e0259672. doi: 10.1371/JOURNAL.PONE.0259672 35202397 PMC8870538

